# Differences in Subjective Well-being Between Older Migrants and Natives in Europe

**DOI:** 10.1007/s10903-016-0537-5

**Published:** 2016-12-10

**Authors:** Gregor Sand, Stefan Gruber

**Affiliations:** Max-Planck-Institute for Social Law and Social Policy, Munich Center for the Economics of Aging (MEA), Amalienstrasse 33, 80799 Munich, Germany

**Keywords:** Immigrant-native gap, Subjective well-being, SHARE, MIPEX, Family reunion

## Abstract

This study examines disparities in subjective well-being (SWB) among older migrants and natives across several European countries using data from the Survey of Health, Aging and Retirement in Europe (SHARE). Our results show a significant SWB gap between migrants and non-migrants that diminishes with increasing age. While migrants from Northern and Central Europe have similar SWB levels as natives, Southern European, Eastern European, and Non-European migrants have significantly lower levels of SWB than the native population. The immigrant-native gap becomes smaller but remains significant after controlling for sociodemographic characteristics and health, the financial situation, citizenship, age at migration, and length of residence. Additionally, we find that the size of the SWB gap varies largely across countries. Current family reunion policies as measured by the Migrant Integration Policy Index (MIPEX) correlate with these country differences. The immigrant-native gap is bigger in countries with restrictive and smaller in countries with open policies.

## Background

Demographic aging and international migration have transformed the European population structure significantly. Many people with migration background have resided in their destination countries for a long time and have become an integral part of society. Given the everlasting flows of migration in- and outside of Europe, the social integration of immigrants has become an important part of research.

A growing body of literature uses either physical/mental health- or well-being-related measures as indicators for social integration [1–8]. Studies on the subjective well-being (SWB) of migrants in later life are scarce and the influence of the institutional conditions of receiving societies on migrants’ SWB has hardly been accounted for. Most studies in this field focus on person-related characteristics. Apart from demographic features (such as gender and age) and migration-specific variables (like length of residence, language skills, and citizenship), they identify economic conditions, health status, social networks, and psychological factors as the main determinants of SWB [9–11]. However, it is important to capture potential influences at the macrolevel as well, especially because immigration policies are very heterogeneous across Europe and because large debates on immigration control and integration policies have been on the political agenda in numerous countries. Yet, there is limited knowledge on how these policies affect the SWB of migrants, particularly in later life.

Conducting research on 63 countries, Bonini finds that 19% of the variation of SWB can be explained by contextual and 81% by individual-specific factors [12]. Two recent studies detect a significant relationship of integration policies with migrants’ self-reported health [2] and SWB [1]. In the latter, Hadjar and Backes find evidence that the SWB gap between migrants and natives is larger in countries with a high GDP and smaller in countries with rather inclusive immigrant integration policies as measured by the Migrant Integration Policy Index (MIPEX).

Our study extends this new strand of research on well-being-related differences between migrants and natives that combines micro- and macrolevel factors using data from the Survey of Health, Aging and Retirement in Europe (SHARE). We contribute to existing research by analyzing the SWB of *older* migrants as a group of increasing importance in the European population structure [13]. As Amit and Litwin [9] point out, the integration of older immigrants has not yet received adequate attention within the literature.

By running our analysis in a cross-national setting we are able to account for *institutional influences* at the macrolevel, thereby focusing on family reunion policies, which turned out to be most influential for SWB among all MIPEX policy areas. Apart from family reunification, the MIPEX also contains the policy areas labor market mobility, education, political participation, access to nationality, long-term residence, and anti-discrimination. As the association with SWB was rather weak for these policy areas, we restrict our analysis to family reunion policies. Especially older migrants who are or will be in need for care might benefit from policies that facilitate family reunification.

“(…) [S]ince the societal SWB level is an indicator of social integration (…)” [1: 646] we assume that integration is achieved once the SWB-levels of migrants and natives are similar [10, 14]. Our main research questions are (a) whether there are any differences in SWB among migrants in relation to the respective native 50+ populations in different European countries, (b) which individual factors play a decisive role in reducing potential group disparities, and (c) if differences in the immigrant-native gap between countries are associated with different family reunion policies.

## Conceptual Framework

The Social Production Function Theory holds that people’s well-being is a function of individual and structural resources and constraints. At the individual level, the most important ones are health, education, income, and social ties [15]. Since the integration process takes place over time and with increasing exposure to the host culture, immigrants’ length of residence in the destination country and their social connectedness have to be accounted for [16, 17]. At the macrolevel, the social infrastructure, laws, regulations, and norms represent key resources and constraints [15]. Assimilation is a process in which laws and institutions play an important role in affecting immigrants’ integration process. According to Sen’s “Capabilities” approach, political and institutional settings limit and structure the opportunities of individuals [18]. The Host Society Environment approach by Maxwell highlights that the geographic variation of integration outcomes depends on the legal situation of immigrants in the place of destination [19]. Access to citizenship and political participation play a major role here. Apart from that, studies have shown that cultural and institutional characteristics inherent to the place of origin (e.g., language proximity to destination country, labor market regulations, education system, transferability of skills and certificates) are crucial for the integration process [10, 20].

Immigrant legislation in Europe is as diverse as its member states. Different policy contexts affect immigrants’ quality of life in various ways and thus the extent to which they feel integrated into the host society [1]. Migrants’ SWB is likely to be afflicted in countries where institutional barriers to achieve social integration are considerably high. Particularly bringing the family together is a major factor promoting the subjective well-being and integration of migrants in their receiving societies [21]. A person’s feeling of comfort and security increases once the family members reside in one place. This is especially the case for older people who are in need of care and support. Among the above-mentioned policy areas, the MIPEX measures the conditions for family reunification across 38 countries including all EU countries. MIPEX is a collaborative study of 25 organizations that was started in 2004 and assigns scores from 0 to 100 for each policy area [22]. High-scoring policy regimes promote the family’s integration in terms of extensive eligibility for family members, manageable requirements for their kin, fairly secure residence status, and sufficient associated rights (e.g., equal access to schools, jobs, housing and social programs). Bureaucratic procedures are quick and free of charge. Low-scoring policy regimes are fairly selective and bureaucratic. They favor migrants with high incomes and stable jobs and implement relatively restrictive procedures for family members in terms of eligibility, conditions, security of status, or associated rights.

In our sample of 11 countries, the country with the highest MIPEX score regarding family reunion policies is Spain with an average value of 87 for the years 2007–2013. Families are allowed to reunite once their sponsor can provide basic housing and legal income based on the general Spanish standards for families. Procedures are fast and more rights-based and secure than in any other country. Eligibility is granted to partners and was expanded to adult children in 2009. While Sweden (79), Belgium (75), and Italy (74) also have comparably high scores, Luxembourg (62), the Netherlands (59), Germany (58), and France (51) range in the middle of the MIPEX classification. Austria (49) and Switzerland (47) belong to the countries with the lowest scores. Only Denmark (36) falls short of them. There immigrants have to wait longer to reunite than in most other developed destination countries. The requirements are highly restrictive including a points-based system, an immigration test, and high fees. Additionally, adult children and parents can only reunite under exceptional circumstances [23–25].

Taking this into consideration, we anticipate the immigrant-native gap in SWB to be more pronounced in countries with restrictive family reunion policies (i.e., low MIPEX family reunion scores) and smaller in countries with more open policies (i.e., high MIPEX family reunion scores).

## Methods

This study uses waves 1, 2, 4, and 5 of SHARE [26]. SHARE was started in 2004 and is a multidisciplinary panel study on health, socioeconomic status, and social and family networks of respondents from 20 European countries plus Israel aged 50 or over [27]. The survey is administered biennially via computer-assisted personal interviews (CAPI). The overall sample comprises more than 120,000 individuals. In order to maintain the maximum number of observations per country, we restrict the sample to all regular SHARE waves and exclude wave 3, which is about respondents’ life histories. We include all migrants (i.e., respondents born in a country other than the country of interview) and natives (i.e., respondents born in the country of interview and having its citizenship) aged 50–85. The observation numbers drop drastically after age 85. Furthermore, we keep all SHARE countries containing at least 100 individual migrants: Austria (AT), Belgium (BE), Switzerland (CH), Germany (DE), Denmark (DK), Spain (ES), France (FR), Italy (IT), Luxembourg (LU), Netherlands (NL), and Sweden (SE). Israel, Greece, Portugal, and the Eastern European states Czech Republic, Estonia, Hungary, Poland, and Slovenia are excluded due to a limited number of migrants and partially very specific migration histories (i.e., Israel, Czech Republic, and Estonia).

Using multivariate random effects (RE) regression models with individual-level clustered robust standard errors we examine differences in SWB between migrants and natives. The dependent variable CASP is a measure for the self-assessed quality of life and well-being of respondents. Quality of life can be operationalized in different ways depending on the field of research (e.g., financial assets in economics or health in medicine). Within the social sciences, good SWB is characterized by a positive state of mind and high levels of life satisfaction [28]. A common instrument to measure SWB is the Satisfaction with Life Scale. However, considering the age structure of SHARE respondents, we opt for CASP, a measure that is designed to quantify the perceived quality of life and subjective well-being of older respondents, initially developed in a population aged 65–75 years [29, 30]. CASP does not only cover aspects of life satisfaction and health, but also social circumstances and functional limitations. It includes questions concerning the domains **c**ontrol, **a**utonomy, **s**elf-realization, and **p**leasure (CASP). SHARE contains an abridged version of CASP that encompasses 12 out of originally 19 items by reducing each of the domains to the three strongest items. In order to do so the statistical analysis used to produce the original 19 item scale was replicated [31]. The score is the sum of all 12 items, which yields a minimum value of 12 and a maximum value of 48. The overall mean in our sample is 38.3 (SD 6.1).

The control variables in this analysis include the following measures: age, sex, marital status, household size (i.e., the number of people per household), number of children, level of education measured by the 1997 version of the International Standard Classification of Education (ISCED), employment status (i.e., retired, employed or self-employed, unemployed, sick, homemaker, other), health (number of chronic diseases), and financial difficulties (original question wording: *Thinking of your household’s total monthly income, would you say that your household is able to make ends meet… (a) with great difficulty, (b) with some difficulty, (c) fairly easily or (d) easily*). We generated a binary variable that equals 1 if the household has great or some difficulty and 0 if the household is able to make ends meet fairly easily or easily.

Our independent variables are the migration-related measures citizenship status, age at migration below/above 18, and length of residence. Apart from these individual factors, we use the average family reunion MIPEX score per country of the period 2007–2013 as macrolevel indicator.

## Results

Descriptive statistics separated by migrants and non-migrants are presented in Table 1. Overall, about 8% of all observations (N = 104,589) in the sample are from respondents born in another country than the one they are living in at the time of interview. Regarding our dependent variable, migrants show on average only a slightly lower CASP value than natives. Comparing the sociodemographic characteristics, we see no striking differences between migrants and natives, with two exceptions: Migrants make up a higher share of people with financial difficulties and, unexpectedly, the educational level measured according to the International Standard Classification of Education (ISCED-97) is slightly higher among migrants. Latter holds for all migrant groups except Southern European migrants (tabulation not shown). Two-thirds of the migrants have the citizenship of the country of residence. They mostly migrated a long time ago. The mean length of residence in the host country is 40.3 years. While the majority of them migrated after the age of 18 or far beyond, one-third moved abroad in their early childhood or adolescence, most likely along with their families. This shows that the migrant population in SHARE is special not only in respect to age but also in the sense that most of the migrants have already been living in the host country for a very long period.

**Table 1:** Descriptive statistics of the sample (natives compared to migrants)

	Natives N (obs.) = 95,940		Migrants N (obs.) = 8649	
	Percent	Mean	Percent	Mean
CASP		38.4 (6.0)		37.9 (6.1)
Age		65.1 (9.0)		63.8 (9.0)
Female	53.9		55.1	
Married/reg. partnership	72.6		69.7	
Household size		2.1 (0.9)		2.1 (0.9)
Number of children		2.2 (1.4)		2.3 (1.5)
Education (ISCED 1997)				
None	11.5		10.4	
Primary level	18.8		13.8	
Lower secondary level	17.0		12.3	
Upper secondary level	29.4		30.1	
Post-secondary non-tertiary	3.3		4.5	
First stage of tertiary	18.7		25.4	
Second stage of tertiary	0.6		1.2	
Employment status				
Retired	51.7		46.2	
Employed/self-employed	29.6		32.9	
Unemployed	2.8		5.4	
Permanently sick/disabled	3.3		4.5	
Homemaker	11.5		9.7	
Other	1.2		1.3	
Financial difficulties	27.3		34.8	
Having chronic disease(s)	60.0		59.2	
Citizenship	100.0		66.6	
Migration after age 18	0		64.1	
Years in destination country		Equal to age		40.3 (17.7)
Total	91.7		8.3	

Table 2 shows the distribution of migrants and their origin regions (i.e., Northern/Central Europe, Eastern Europe, Southern Europe, and non-European areas) across all destination countries. For 85 migrants the information on region of origin is missing. The table shows that the distribution of all migrant groups is very heterogeneous across countries, which makes it necessary to control for country fixed effects in our regression models. Overall, migrants from Northern/Central Europe immigrating to other countries in Northern and Central Europe (DK, CH, SE, and BE) are the largest group with 36%, followed by non-Europeans with 31%. Especially non-European migrants might exhibit lower levels of SWB because high institutional barriers can hamper their social integration (e.g., legal access to labor market depending on citizenship). A closer look at the countries with the highest share of non-European migrants shows that in NL they are mainly from Indonesia and the former Dutch territories in the Caribbean, in FR and IT mainly from Northern Africa, and in ES mainly from Latin America and Morocco (not shown here). Both migrants from Southern and Eastern Europe make up about 16% in total, with the former representing the highest share in Luxembourg (mainly from Portugal) and the latter being the largest group in Austria and Germany (mainly from former Yugoslavia, former Czechoslovakia, and Poland).

**Table 2:** Distribution of migrants’ origin regions by destination country

Country	Northern/Central Europe	Eastern Europe	Southern Europe	Non-European	Number of observations
Austria	34.5	**39.3**	9.2	17.0	882
Germany	31.3	**37.4**	7.9	23.4	1342
Netherlands	22.8	3.2	6.3	**67.7**	505
France	14.7	3.7	23.6	**57.9**	1265
Denmark	**48.2**	7.7	4.5	39.6	311
Switzerland	**54.5**	11.3	21.2	13.0	1301
Sweden	**60.4**	17.9	3.2	18.5	853
Spain	18.4	10.2	3.3	**68.1**	392
Italy	28.2	7.1	12.2	**52.6**	156
Belgium	**38.5**	4.3	30.3	26.9	1055
Luxembourg	39.7	5.0	**43.5**	11.9	504
Total *N*	3120	1356	1406	2682	8564
Total %	36.4	15.8	16.4	31.3	100.0

All numbers in bold represent the main origin region of migrants per destination country

**Fig. 1:**
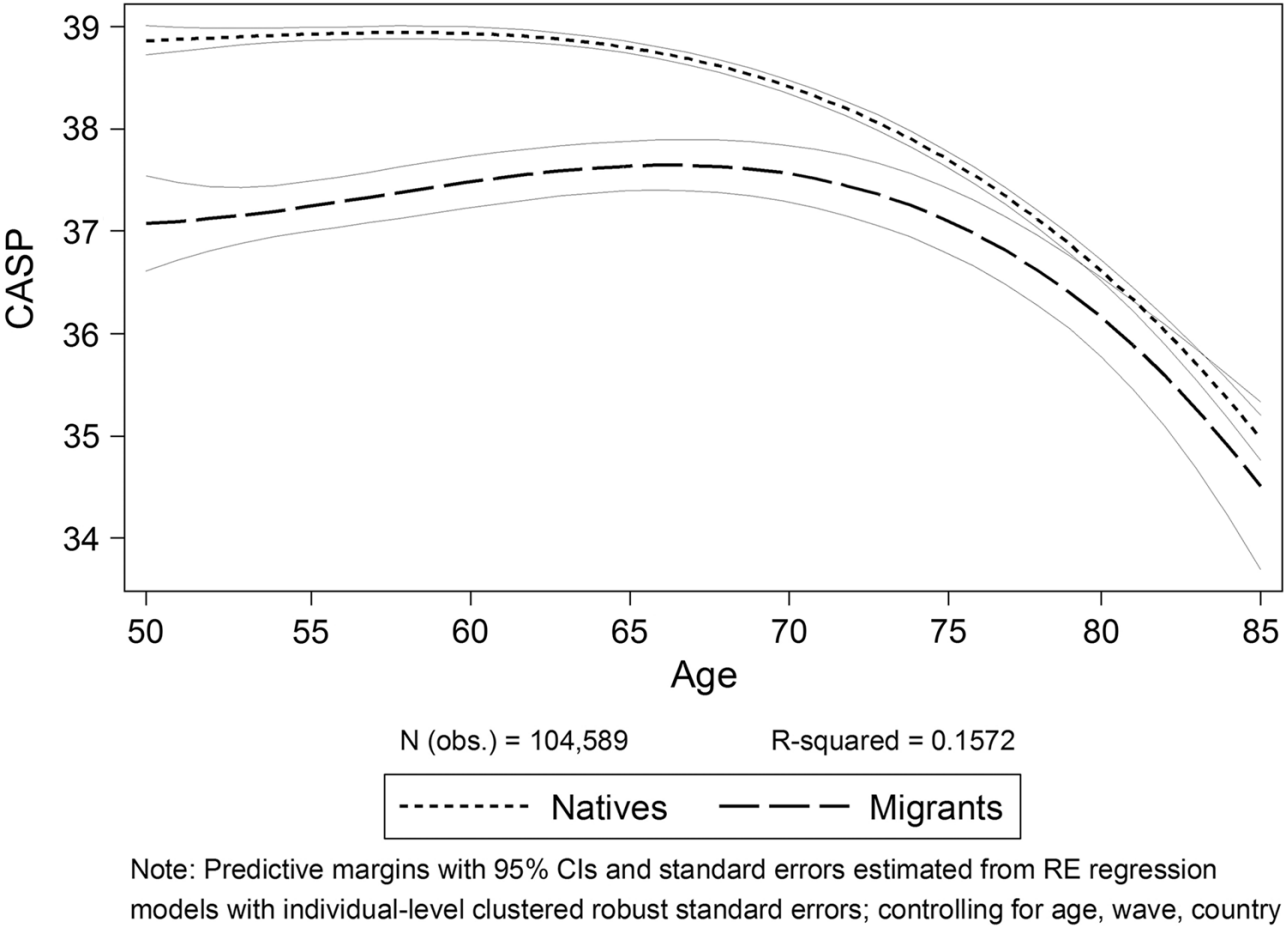
CASP for natives and migrants by age

As the first step of our analysis, we explore the differences in SWB between migrants and natives by running random effects regression models to estimate group-specific growth curves controlling for age, time of interview (wave), and country. In Fig. 1, it can be seen that within the older population and compared to natives, migrants show significantly lower levels of subjective well-being. The differences decrease with increasing age and become statistically insignificant beyond the age of 78.

**Fig. 2:**
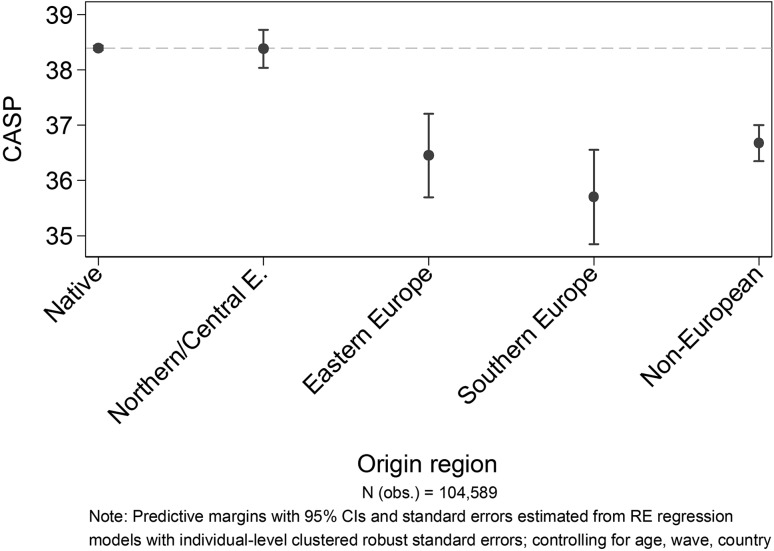
Predicted values of CASP by migrants’ origin region (reference: natives)

Figure 2 displays the immigrant-native gap by origin regions. The horizontal line represents the CASP level of non-migrants. For Northern/Central European migrants no significant differences can be observed. Their SWB level is almost equal to the one of natives. Eastern European, Southern European, and non-European migrants show CASP levels that are significantly lower than the levels of the native population. Surprisingly, the gap is largest for Southern European and not—as expected—for non-European migrants.

**Fig. 3:**
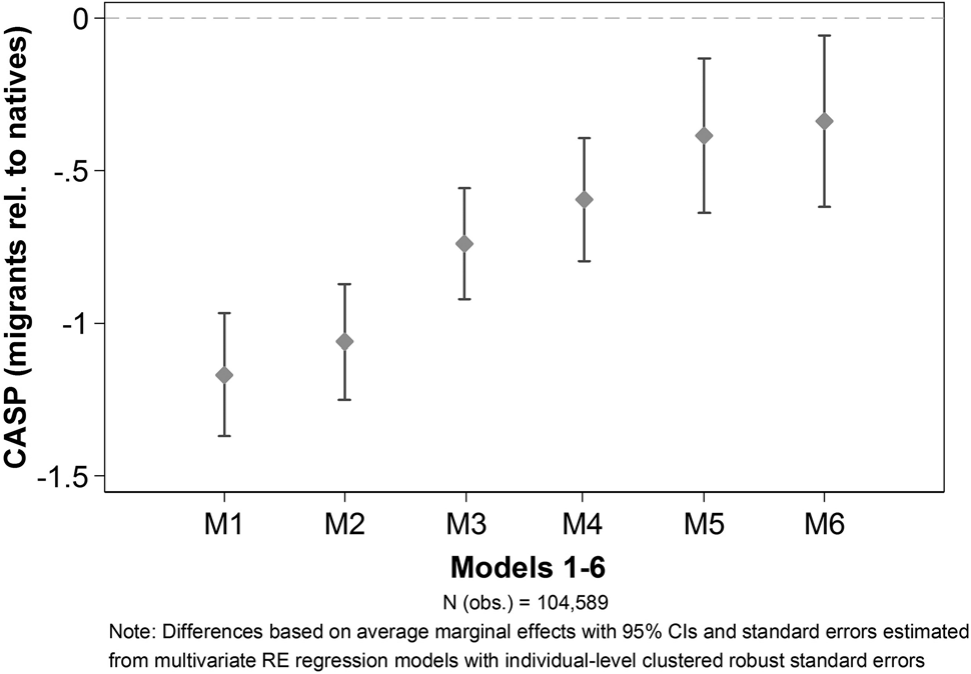
Regression models 1–6 for CASP

Next, we examine individual factors that may have an impact on reducing the immigrant-native gap by estimating multivariate random effects regression models. As illustrated in Fig. 3, we start with a basic model (M1) controlling for age, time of interview (wave), country and then stepwise add additional control variables: sociodemographic characteristics and health (M2) and having financial difficulties (M3). Then we add our independent variables: having the citizenship of the country of residence (M4), having migrated before/after the age of 18 (M5), and finally length of residence (M6; for natives the latter equals age). It can be observed that each model contributes to explaining the variation in SWB between migrants and natives. While sociodemographic characteristics and health (M2) do not show large effects, the gap becomes considerably smaller after accounting for the financial situation (M3), having the citizenship of the country of residence (M4), and having migrated before the age of 18 (M5). The years migrants have resided in the destination country (M6) slightly contribute to reducing the gap. After all, even after controlling for all individual characteristics in the full model, the immigrant-native gap remains significant.

**Fig. 4:**
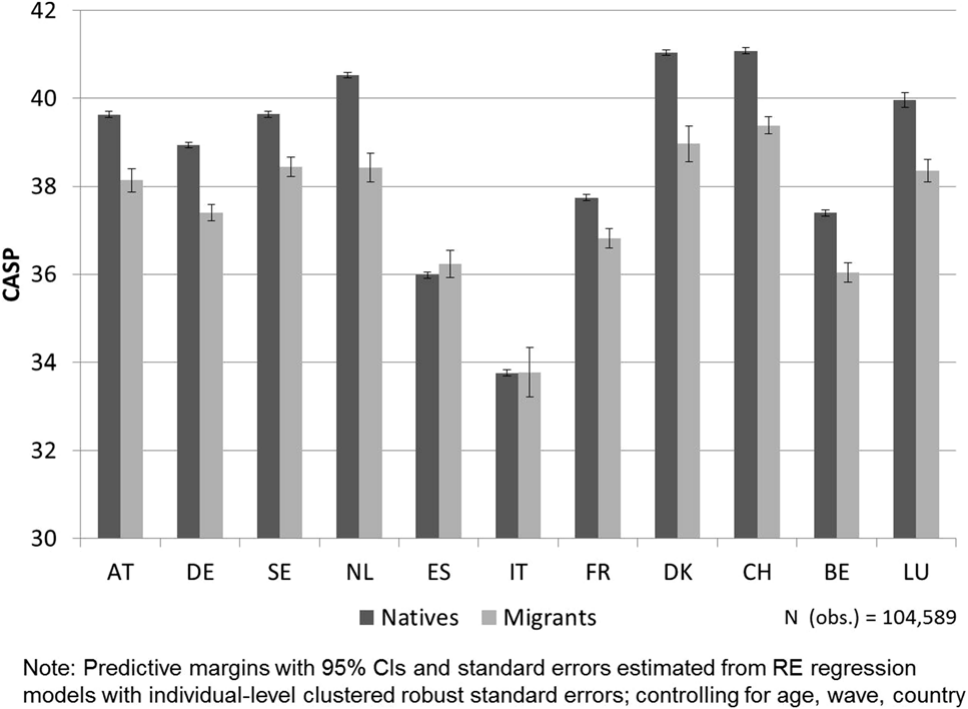
Predicted values of CASP for natives and migrants, by country

By moving our analysis to the country level, we first analyze the group differences between countries by controlling only for age, time of interview (wave), and country. The predictive margins in Fig. 4 illustrate that there are large variations concerning the size of the immigrant-native gap across countries. Migrants have a lower level of SWB than the respective native population in all countries with the exceptions of ES and IT. The differences are largest in NL and DK.

**Fig. 5:**
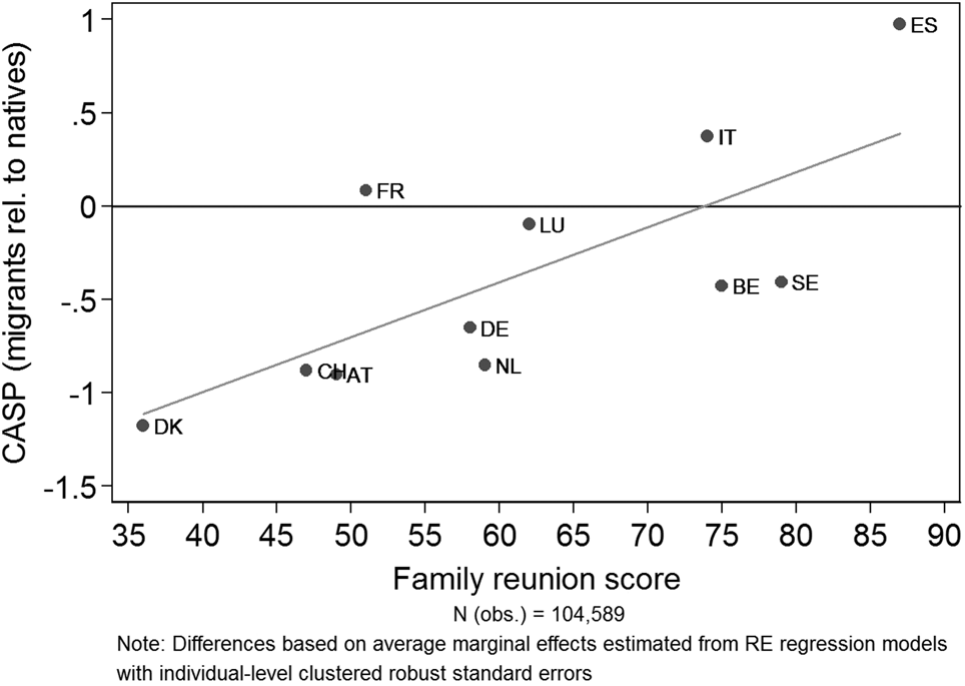
Country correlation matrix of the immigrant-native gap in CASP and the MIPEX family reunion score

Since we observe great variation in terms of integration policies in Europe, we complete our analysis by exploring to what extent the country disparities are associated with their institutional framework. Controlling for all individual factors (M6), Fig. 5 plots the differences in SWB of migrants relative to natives (y-axis) against the country-specific average score in the MIPEX policy area family reunion (x-axis). The horizontal zero line represents the SWB level of natives. The slope of the graph clearly shows a positive association with family reunion policy context. The immigrant-native gap is comparably large in countries with low MIPEX scores (i.e., rather restrictive family reunion policies) and becomes smaller among countries with higher scores (i.e., more open family reunion policies). For instance, controlling for all individual factors, the CASP score of migrants in DK is on average one CASP point lower than the one of natives, whereas in ES it is one CASP point higher than in the native reference group.

The results turned out to be robust after running our analyses separated by gender and by replacing CASP with life satisfaction as a quality of life measure (not shown here).

## Discussion

The present study focuses on older migrants and explores the differences in SWB between migrants and non-migrants in different European countries. While most studies employ only individual variables, our analysis also integrates institutional factors by including policy context in terms of family reunion policies. Apart from destination effects, we also account for origin effects by examining the role of migrants’ region of origin. The major findings of this study are specified in the following paragraphs.

We detect significant differences in SWB between older migrants and non-migrants that decline with increasing age. While SWB differences are starker for migrants originating from Southern and Eastern Europe as well as for non-European migrants compared to native born, the SWB levels of migrants from Northern and Central Europe are comparable to those of non-migrants. This is consistent with the results by Kämpfer [10] who finds significant differences between migrants and natives for Germany and identifies migrants from Southern Europe as well as from Turkey and former Yugoslavian countries as the groups with the lowest SWB levels.

Moreover, the immigrant-native gap in SWB does not diminish largely after adding socioeconomic status and health, which belong to the key correlates of SWB [32, 33]. This may have to do with the fact that the migrants and non-migrants in our sample do not vary largely with regard to sociodemographic characteristics and health. Material resources strongly contribute to SWB and social integration [34]. Our data suggest that having no financial difficulties significantly diminishes the immigrant-native differences in SWB. Apart from that, migration-related factors help reducing the group disparities: While Tucci et al. [11] find that citizenship does not play an important role in reducing the SWB gap in Germany, our findings show that having the citizenship of the destination country reduces the SWB gap for migrants. Additionally, having migrated at an early age and the length of residence in the host country turn out to be important factors. Young migrants who grew up and were educated in the destination societies and migrants who have resided in their host countries for a considerable amount of time tend to be better assimilated than migrants who arrived recently and/or at later ages. This is in accordance with the empirical findings formulated by Gordon [16] and Berry et al. [17].

On the country level we observe considerable variation across countries regarding the size of the SWB gap. This variation is correlated with institutional context: The more open and inclusive a country’s family reunion policy, the smaller the SWB gap for migrants. The findings are in line with the Capabilites approach by Sen [18] and the Host Society Environment approach by Maxwell [19] who stress the importance of structural conditions for promoting integration. They are also consistent with Hadjar and Backes [1] who detect a positive correlation between the overall MIPEX score and SWB.

Nevertheless, some limitations should be considered when interpreting these results. The migrant population in SHARE is special because it includes migrants aged 50 years and older who stayed in their destination countries and speak the corresponding language proficiently. Considering that low levels of SWB might be a reason to return to the country of origin [35] and that language skills are a major source of social integration [16, 36], the SWB levels of the migrants in our sample might be biased upwards. Apart from younger migrants who are not part of the sample, we excluded respondents aged 85 and above due to low case numbers. Future research should examine whether the results also hold for younger migrants and the oldest old. Apart from that, Diener [37] pointed out that personality-related variables (e.g., self-esteem) play a role for the individual SWB. Since this information is not part of the data, we could not account for personality characteristics.

Concerning policy implications, our results indicate that migrants’ SWB can be improved by (a) providing the preconditions for equal access to economic resources, by (b) streamlining naturalization and citizenship regulations, and by (c) fostering an integrative receiving context. Promoting the family’s integration in terms of easy access and sufficient associated rights for family members increases migrants’ SWB and facilitates their social integration. Sponsoring the reunification of family members is especially meaningful for older migrants who are or will be in need for care. In the long run, this helps relieving the social security systems of the destination countries and strengthens social cohesion.
